# Acidic pH environment induces autophagy in osteoblasts

**DOI:** 10.1038/srep46161

**Published:** 2017-04-06

**Authors:** Zhichao Zhang, Qingguo Lai, Yanan Li, Chao Xu, Xiaopeng Tang, Jiangbo Ci, Shaolong Sun, Bingbing Xu, Yan Li

**Affiliations:** 1 Department of Oral and Maxillofacial Surgery, the Second Hospital of Shandong University, Jinan, China; 2 School of Stomatology, Shandong University, Jinan, China; 3 School of Medicine, Shandong University, Jinan, China

## Abstract

Osteoblasts (OBs) play an important role in bone fracture healing, yet the extreme adverse microenvironment in fracture sites has a negative impact on the survival of OBs. Therefore, it is important to study how OBs behave in the complex fracture microenvironment. Studies have shown that autophagy plays a pivotal role in maintaining cellular homeostasis and defending the cell against adverse microenvironments. In this study we found the induction of autophagy in OBs at femoral bone fracture sites, which may be a result of ischemia, oxidative stress and hypoxia within the local area. At fracture sites a low pH environment also developed. Until now it has been unclear whether the induction of autophagy in osteoblasts is triggered by the acidic pH environment. Therefore, we cultured OBs *in vitro* in media of different pH values, and found both autophagy and apoptosis increased in OBs in acidic conditions. However, when autophagy inhibitor chloroquine (CQ) was used, apoptosis increased significantly compared with that without CQ. Thus indicating that inhibition of autophagy may promote apoptosis in OBs in an acidic environment, which may provide a new therapeutic strategy to decrease cell apoptosis in OBs through the use of drugs that modulate the autophagic state.

Physical trauma including bone fractures are accountable for an increasing proportion of the global disease burden^1^,^2^ posing a major socioeconomic, clinical, and scientific challenge. Survival of patients after severe tissue trauma depends on an efficient molecular and cellular responses and effective regeneration of the damaged tissue. Bone fracture healing is a complex process, initially the soft (cartilage) callus forms, followed by formation of the hard (bone) callus. Although most fractures heal routinely, about 5–10% of fractures fail to heal normally and are described as displaying bone nonunion^3^. Many factors contribute to the pathogenesis of a delayed union or bone nonunion. It is known that osteoblasts (OBs), the cells responsible for bone formation, play a significant role^3^. The extreme adverse microenvironment in fracture sites has a negative effect on the survival of OBs, which may be a key cause for bone nonunion and segmental bone loss after fractures. As such, it is of clinical significance to study how the osteoblasts react in the complex fracture environment.

Autophagy is a catabolic process of eukaryotic cells in which cellular components, such as damaged macromolecules and organelles, are degraded by the lysosome in order to maintain cellular homeostasis^4^,^5^,^6^. Three major forms of autophagy are observed in mammalian cells, namely: chaperone-mediated autophagy, microautophagy, and macroautophagy. Macroautophagy (referred to hereafter as autophagy), the most widely studied form, acts in concert with the ubiquitin-proteasome system (UPS) to maintain cellular homeostasis^7^. The cytoplasmic material including damaged organelles, intracellular pathogens and protein aggregates are enclosed by double-membraned vesicles known as autophagosomes. Autophagosomes are delivered to lysosomes where upon their contents are degraded after fusion. These catabolic products can re-enter bioenergetic and/or anabolic process^8^.

During physiological conditions, autophagy plays an important role in removing defective organelles or protein aggregates. However, when cells are exposed to stressful conditions, autophagy is induced to degrade cellular components in order to provide an energy source^5^,^6^. Some reports have shown that hypoxia, serum deprivation and oxidative stress induce autophagy and promote the survival of mesenchymal stem cells or OBs^9^,^10^,^11^,^12^,^13^. However, acidic pH environments also develop in fracture sites^14^,^15^ and the role of these acidic environments in the induction of autophagy in OB’s is still unclear.

After bone fracture, the accumulation of lactic acid may occur as the result of an interruption in blood supply in the local environment. Both hypoxic and ischemic conditions can cause extracellular acidosis with low pH, consequently an acidic local environment can develop at the fracture site^14^,^15^. The application of tissue engineering can also lead to local acidosis during the induction of ossification^16^,^17^. This local acidosis can lead to the cell death of osteoblasts, which results in bone loss^18^,^19^,^20^,^21^,^22^. Osteoblasts may persist initially in a low pH environment before undergoing cell death, yet how the osteoblasts react in this environment is still not well understood. Therefore, we hypothesized that autophagy may be directly induced in OBs when challenged by an acidic environment and autophagy may play a pivotal role in the protection of OBs from apoptosis in such conditions.

In this study, autophagy was first detected at bone fracture sites *in vivo*. Subsequent *in vitro* study was conducted to investigate autophagy and apoptosis of OBs in cultured in media of different acidities. It is proposed that modulation of the autophagy state may provide a new therapeutic strategy to promote bone healing.

## Results

### Autophagy is induced in OBs at fracture sites

**Figure 1:**
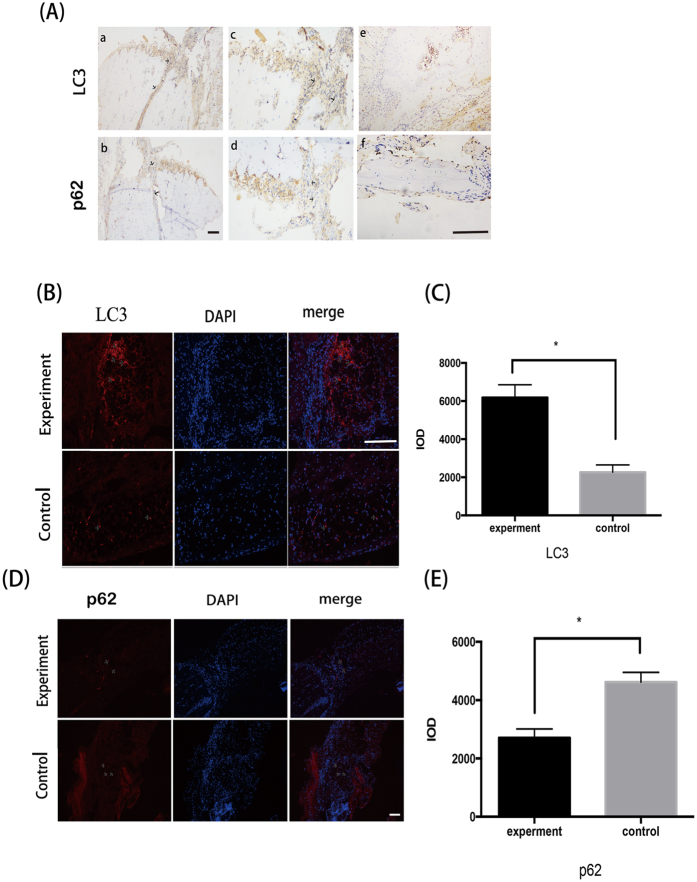
Autophagy is induced in OBs near fracture sites in the group 36 h after fracture. Longitudinal sections of rat fractured bones were detected. (**A**) Immunohistological staining showed the expression of LC3 and P62 in OBs at fractured sites (arrows). Size bar = 100 μm. As showed in figures e and f were control groups, a and b were experiment groups. a and c were from the same section with different magnification which showed the expression of LC3. b and d were from the same section with different magnification which showed the expression of P62. (**B**,**D**) Immunofluorescence analysis noted the distinct punctate pattern of LC3 and P62 expression (arrows) in OBs near fracture sites after 36 h. Size bar = 100 μm. (**C,E**) Quantified analysis of LC3 and P62 expression in osteoblasts in bones from the control and fracture group. *P < 0.05 vs.control. Values are the mean ± sd, *P < 0.05 in comparison to the control by one-way ANOVA.

Positive immunohistological or immunofluorescent staining was not found in group A(6 h) and group B(24 h). Immunohistological examination of femora 36 hours post fracture in group C, showed that LC3 immunoreactivity could be detected in a punctate pattern in OBs at fracture sites, which was not observed in un-fractured controls (Fig. 1A). Similarly, in group C immunofluorescent staining of LC3 also showed high expression in OBs around the bone marrow cavity in the fractured areas compared with the control femora, suggesting that autophagy was induced in OBs at fracture sites (Fig. 1B).

### Low pH media decreases cell viability and induces apoptosis in OBs

**Figure 2:**
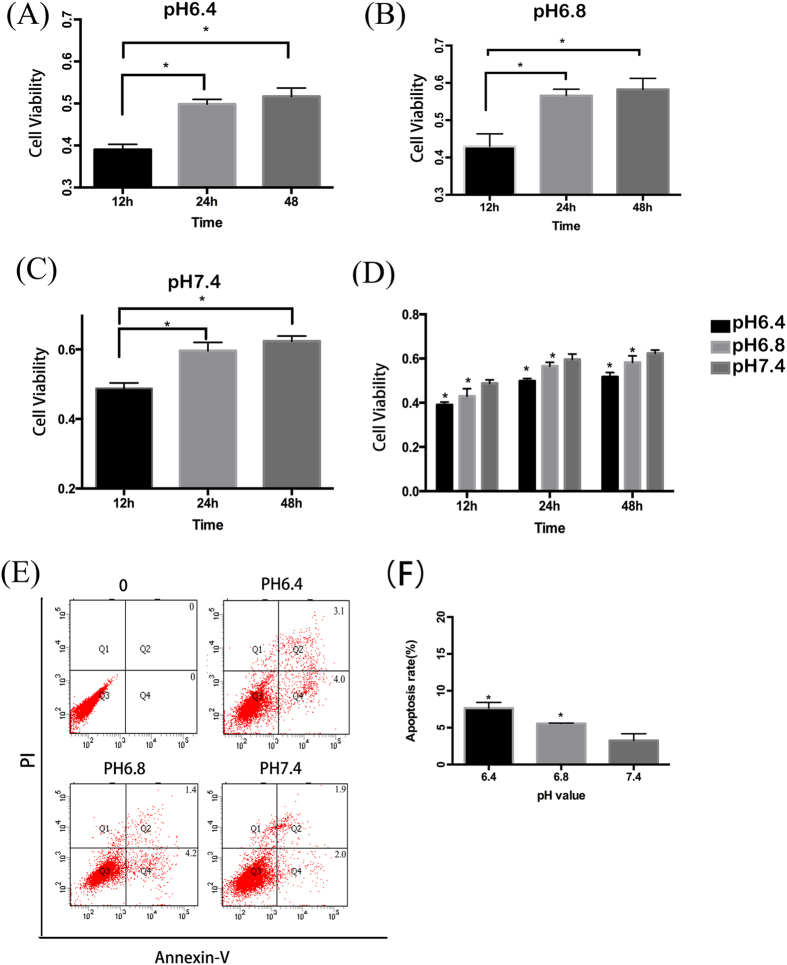
Acidic pH environment has a negative effect on the survival of OBs. OBs were cultured in media with different pH values for 12, 24, 48 h and cell viability was measured by MTT. Apoptosis was determined by flow cytometry. (**A**–**D**) MC3T3-E1 cells was cultured in media with pH6.4, 6.8 and 7.4 for 12, 24, 48 h, and cell viability was detected. *P < 0.05 vs.12 h with pH6.4 and 6.8, and *P < 0.05 vs.6 h with pH7.4. (**E**) Cell apoptosis increased in media with pH 6.8 and 6.4 at the time of 48 h including both early apoptosis and late apoptosis. (**F**) Analysis of apoptotic rate with pH 6.4, 6.8 and 7.4 at the time of 48 h. *P < 0.05 vs.pH 7.4. Values are the mean ± sd, *P < 0.05 in comparison to the control by one-way ANOVA.

Viability of osteoblastic cell line, MC3T3-E1, was markedly influenced by the acidity of the culture media in which they are grown. Both pH values of 6.4 and 6.8 induced a significant decrease in cell viability compared with that of pH 7.4 (P < 0.05). While all cells died when cultured in media at pH 6.0. Furthermore, a pH- and time-dependent decrease in cell viability was observed, with a significant difference in viability between pH value of 6.4 and 6.8 (Fig. 2A–D). Levels of apoptosis were determined by flow cytometry utilising Annexin-V and PI dual labelling. Cells in the Q2 and Q4 quadrants represent apoptotic cells. Increased rate of primarily early apoptosis (but also some late apoptosis) was detected in MC3T3-E1 cells in media with pH 6.8 and 6.4 after 48 h of exposure. Compared with cells cultured at pH 7.4, those at pH 6.4 and 6.8 exhibited statistically significantly increased levels of apoptosis (P < 0.05). (Fig. 2E,F). Comparison between the levels of apoptosis between the two acidic culture conditions showed increased apoptosis at pH6.4, however there is no statistically significant difference (P > 0.05).

### Low pH media induces autophagy in OBs

**Figure 3:**
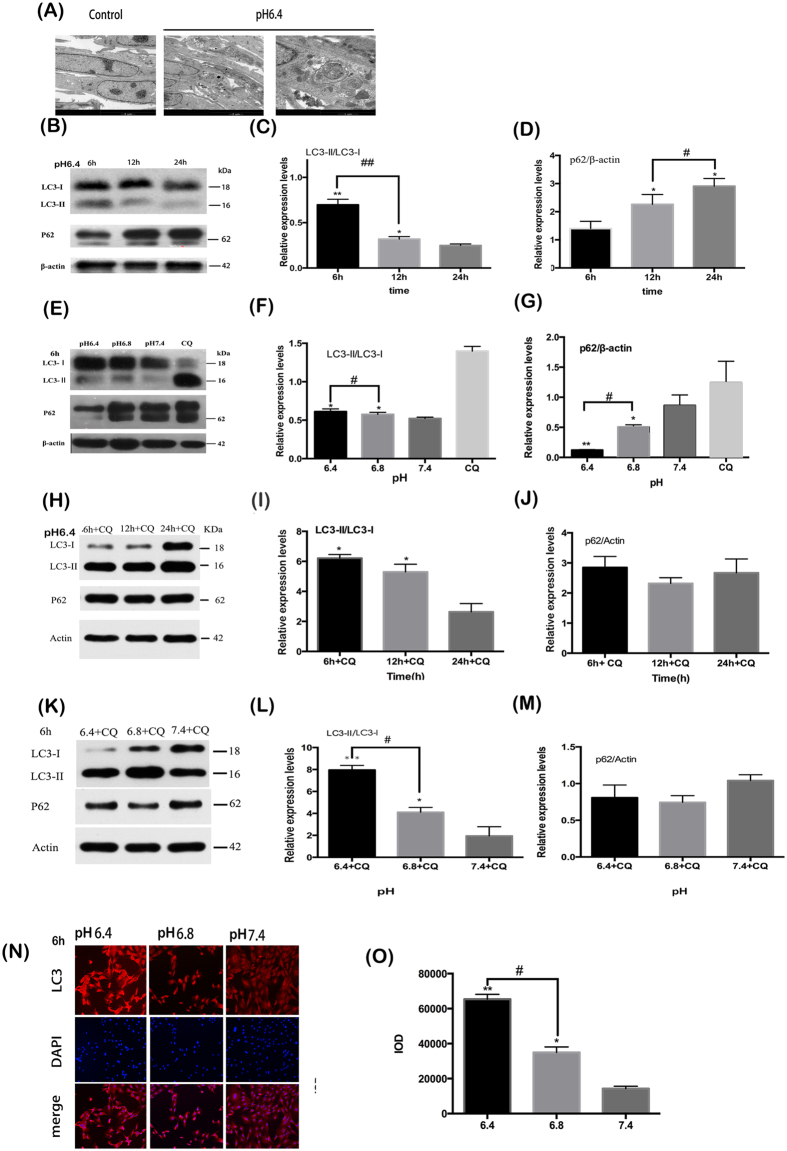
Acidic pH environment induces autophagy in murine osteoblastic cell line MC3T3-E1. (**A**) More autophagosomes were observed in the media of pH 6.4 with typical double-membrane structures surrounded with cytoplasm and mitochondria. (**B**)The expression of LC3-II and P62 in OBs as the treatment time in pH 6.4 is prolonged. (**C**) Densitometric analysis of LC3-II/LC3-I at 6 h, 12 h and 24 h. **P < 0.01 vs.24 h, *P < 0.05 vs.24 h. (**D**) Densitometric analysis of P62/β-actin at 6 h, 12 h and 24 h. *P < 0.05 vs.6 h. (**E**) The expression of LC3-II and P62 in OBs as the pH value decreased for 6 h. (**F**,**G**) Densitometric analysis of LC3-II/LC3-I and P62/β-actin with pH 6.4, 6.8 and 7.4 for 6 h. *P < 0.05 vs. pH 7.4. (**H**) The expression of LC3-II/LC3-I and P62 in OBs in pH 6.4 with CQ as the treatment time was prolonged. (**I**,**J**) Densitometric analysis of LC3-II/LC3-I and P62/β-actin at 6 h, 12 h and 24 h in pH 6.4. *P < 0.05 vs.24 h + CQ. (**K**) The expression of LC3-II/LC3-I and P62 in OBs as the pH value decreased for 6 h. (**L**,**M**) Densitometric analysis of LC3-II/LC3-I and P62/β-actin among 6.4 + CQ, 6.8 + CQ and 7.4 + CQ for 6 h. **P < 0.01 vs.pH 7.4 + CQ, *P < 0.05 vs. pH 7.4 + CQ. (**N**) Immunofluorescence staining of LC3 were labelled red and nuclei were stained with blue. Size bar = 100 μm. The brightness of red around nuclei increased with the decrease of pH value in media. (**O**) Quantified analysis of immunofluorescence staining of LC3. **P < 0.01 vs.pH 7.4, *P < 0.05 vs. pH 7.4. Values are the mean ± sd, *P < 0.05.**p < 0.01, in comparison to the control by one-way ANOVA.

Transmission electron microscope (TEM), is the gold standard for the detection of autophagy. TEM was carried out to confirm the existence of autophagy in the osteoblastic cells cultured in acidic media. As shown in Fig. 3A, the morphology of the cells was changed after exposure to low pH media, with more autophagosomes observed in the pH 6.4 and 6.8 conditions. The typical double-membrane structures surrounded with cytoplasm and mitochondria were found primarily in the cells cultured in an acid environment.

In order to determine whether an acidic environment increases autophagy in osteoblasts, expression level analysis of autophagy marker protein LC3, which can be converted from LC3-I to LC3-II, was undertaken. Under prolonged exposure to acidic conditions, the ratio of LC3-II/LC3-I in OBs decreased (Fig. 3B,C). Western blot analysis showed that after 6 h of cell culture, an inverse correlation was observed between the increasing expression of LC3-II in OBs and decreasing pH of the media(Fig. 3E,F). Autophagy is a highly dynamic process; detection of P62, the substrate of autophagy, is utilised to monitor autophagy flux. Contrary to the LC3-II, P62 expression increased gradually with treatment time prolonged and the increase of pH(Fig. 3B,D,E,G). Our results suggested that reduction of environmental pH increased the expression of LC3-II and reduced the levels of P62 (Fig. 3B–G). Furthermore, OBs cells exposed to low pH and treated with CQ were also studied. The ratio of LC3-II/LC3-I in pH 6.4 with CQ decreased as the treatment time is prolonged and the increse of pH (Fig. 3H–M). In addition this study has shown the cellular localisation of LC3 is restricted to in the cytoplasm of osteoblasts under acid environment (Fig. 3N,O).

### Suppression of autophagy promotes apoptosis in OBs

**Figure 4:**
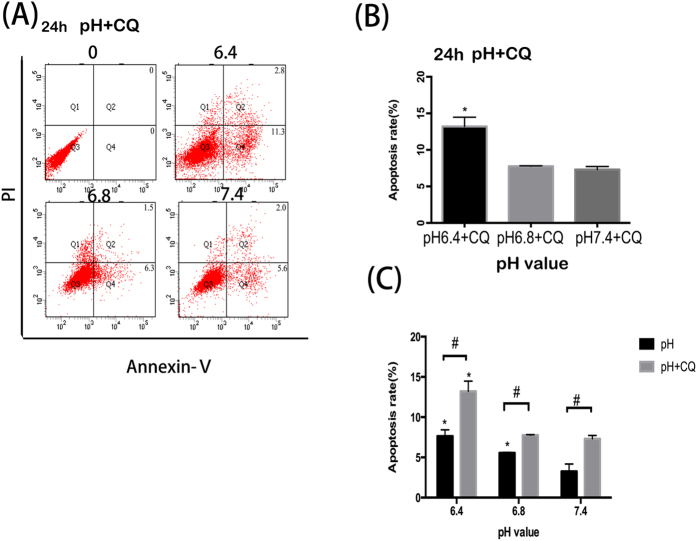
Suppression of autophagy promotes apoptosis in OBs. (**A**) Cell apoptosis increased in media with CQ with the decrease of pH value at the time of 24 h. (**B**) Cell apoptosis rate with 6.4 + CQ, 6.8 + CQ and 7.4 + CQ for 24 h. *P < 0.05 vs. pH 6.8 + CQ(or pH 7.4 + CQ). (**C**) Moreover, the rate of apoptosis in OBs with CQ is higher than that without CQ. Values are the mean ± sd, *P < 0.05 in comparison to the control by one-way ANOVA.

In order to study the relationship between apoptosis and autophagy, MC3T3-E1 cells were exposed to autophagy inhibitor CQ. The rate of cellular apoptosis increased when cells were treated with CQ in parallel with exposure to acidic conditions (Fig. 4A,B). Moreover, a larger proportion of apoptotic cells were observed when the cells were treated with CQ at all three experimental pH levels (7.4,6.8 and 6.4) (Fig. 4C).

## Discussion

Low tissue pH and locally acidic environments develops at bone fracture sites or in tumours, as a result of hypoxic or ischemic conditions, and poses a deleterious environmental stress. The induction of autophagy has been increasingly observed in stem cells under serum deprivation, oxidative or hypoxic stress and radiation insult^4^,^9^,^10^,^11^. However, whether autophagy is directly induced in low acidic situations is still unknown and the connections between apoptosis and autophagy in OBs is still unclear.

Previous study has shown osteocytes can display a punctate distribution of LC3 in both rats and human cortical bone. However, particulate LC3 fluorescence has not been observed in bone-lining cells or osteoblasts. Furthermore, autophagic osteocytes were primarily observed residing at a distance from the haversian canal^23^,^24^. Our study found the expression of LC3 in osteoblasts which indicated that osteoblasts may undergo autophagy at bone fracture sites. Given the acidic environment caused by hypoxic and ischemic conditions at the bone fracture site we postulated that osteoblasts may be induced to autophagy through extracellular acidosis and low pH.

Thus, we designed *in vitro* experiments to investigate the levels of autophagy in OBs cultured in media of a variety of pH. In the present study, autophagosomes were observed in OBs in media of pH 6.4 and 6.8 using TEM. Autophagy decrease was detected with prolonged exposure to low pH, by examining the autophagy-reliable marker LC3. Meanwhile, the expression of LC3-II in OBs increased when cells were grown in decreasing pH conditions. Furthermore, LC3-II accumulated greatly with the inhibition of lysosomal degradation and autophagic flux, through the administration of CQ^25^,^26^. Autophagy is a highly dynamic process. The amount of LC3-II detected by western blot (WB) during the process of autophagy, reflects synthesis as well as consumption of the protein by the lysosomal compartment. In addition to protein levels of LC3, P62 is an important marker to study autophagic flux^27^,^28^,^29^,^30^. P62 is exclusively degraded after being bound directly to LC3 by the autophagy-lysosome pathway^31^,^32^. Therefore, P62 decreases after autophagy is induced and accumulation of P62 protein level can be detected when autophagy is inhibited^27^,^28^. Thus, in this study P62 was also examined to detect the dynamic autophagic flux. Our data shows that P62 detection by western blot exhibits two bands which may result from phosphorylation of P62.

Paradoxically, autophagy is closely linked to both cell survival as well as the induction of the death process. However, the relationship between autophagy and apoptosis is still not well understood^33^,^34^. Many studies have reported the complex relationship between apoptosis, autophagy and necrosis with different conclusions^35^. There is not a consensus over the relation between autophagy and apoptosis^36^,^37^,^38^,^39^,^40^. Autophagy is a highly controlled catabolic mechanism for the removal of dysfunctional or unwanted proteins and organelles, which plays an important role in protective process in various stress environment^41^,^42^. However, this study found that apoptosis was promoted and cell viability decreased with increasing acidity. Furthermore, levels of apoptosis also increased when autophagy inhibitor CQ was used, which indicates inhibition of autophagy may promote apoptosis in OBs when in acidic environment. Autophagy plays many roles in regulating cell survival and cell death. It can convert from a defensive mechanism against environmental stimuli, to a cell death pathway when conditions change^43^,^44^. Our findings suggest that culture of OBs in low pH media may activate the intrinsic protective autophagic process, as such the cells will be more tolerant to acidic stress. However, if the low pH is far beyond the tolerance of OBs or the induced autophagy is supressed, the cells are more easily to undergo apoptosis.

In summary, we found for the first time that autophagy is induced in bone fracture sites *in vivo* and that acidic culture conditions enhanced autophagy in OBs *in vitro*. We also present that co-treatment with autophagy inhibitor CQ renders cells more sensitive to apoptosis. However, in future additional experiments are needed to confirm that autophagy plays the protective role in helping OBs survive in an acidic environment. These results put forward a new understanding of low pH environments and the process of autophagy in OB. These findings may provide a new strategy to promote bone fracture healing in low pH tissue environments through the use of drugs that modulate the autophagic state.

## Methods

### Animal studies

Six-week-old male KM rats were used in this experiment. The body weights ranged from 30 g to 40 g at the beginning of the experiment. Twenty-seven KM rats underwent fracture of the right femora and were then randomly assigned into 3 groups of 9 (group A,B and C). The rats were treated with anaesthesia by intraperitoneal injection of 10% chloral hydrate. All animals receive no treatment after the bone fracture models were established. The animals from group A, B and C were humanely killed with sodium pentothal injections 6 h, 24 h and 36 h post-fracture, respectively. All fractured femora were harvested and the left femora were also extracted to act as a non-fractured control. The specimens were fixed with 4% paraformaldehyde and decalcified in 10% EDTA solution which changed every 4 days at 4 °C, then subjected to graded ethanol dehydration with xylene, and embedded in paraffin wax. We conducted immunohistochemical and immunofluorescence staining for LC3 and P62. Samples were observed and imaged by microscope (Carl Zeiss Canada).

All animal studies were carried out under the guide of the Care and Use of Laboratory Animals of the Chinese Science and Technology Ministry. This experiment was approved by the Committee on the Ethics of Animal Experiments of the Second Hospital of Shandong University (Permit Number: KYLL-2016LW-0016).

### *In vitro* cell culture

Murine osteoblastic cell line, MC3T3-E1, was cultured in α-minimal essential media (α-MEM), supplemented with 10% fetal bovine serum (FBS), containing 100 units/ml penicillin and 100 μg/mL streptomycin, and maintained at 37 °C humidified atmosphere of 5% CO_2_. The media was changed every 3 days. The pH value of media was adjusted by adding 5 N HCL and 5 N NaHCO, and 20 m M HEPES were added to α-MEM_3_ in order to avoid pH variations. Before each experiment, the acidic pH media was measured and filtered to ensure consistency of the experimental conditions.

### MTT assay for cell viability

In order to determine whether the acidic environment has a negative impact on cell proliferation, cells were exposed to different pH media for 12, 24, 48 h, before cell viability was measured by MTT assay as previously described^45^. Osteoblasts were seeded into 96-well plates with a density of 5 × 10^4^ cells/well. After being incubated for 24 h, the cells were replaced with media of different pH (6.0,6.4,6.8, and 7.4) at indicated times. Then 10 μL of MTT stock solution (5 mg/ml) was added into the wells. After being incubated in the dark at 37 °C for 4 h, 100 μL DMSO was added into the well. The absorbance was measured at 490 nm with a bio-tek 311 microplate plate reader (elx 800, bio-tek, Winooski, vt).

### Apoptosis detection by flow cytometry

Annexin V (a sensitive indicator for detection of early apoptosis) and propidium iodide (PI) (a sensitive indicator for detection of late apoptosis), were used to detect apoptosis in obteoblasts. The experiment was divided into two groups (A and B). In group A, osteoblasts were cultured in media of different pH values (6.4,6.8 and 7.4). In group B before cells were exposed to media with same pH values as group A, CQ was added to inhibit autophagy. Cellular apoptosis was measured by the Annexin V-FITC Apoptosis Detection Kit. Cells were plated in 6-well plates and maintained at 37 °C for 24 h, and then treated with media of different pH value (6.4,6.8, and 7.4). Cells were harvested and washed with cold PBS gently for three times after 48 h, and then resuspended in 100 μL Binging Buffer. Annexin V-FITC and PI were added and the cells were incubated for 15 min in the dark. 400 μL Binging Buffer was then added and the cells were analysed by flow cytometry (Accuri C6 Cytometer, BD Biosciences, San Jose, USA).

### Transmission electronic microscopy

After being treated with media of different pH (6.4,6.8 and 7.4), the cells were prefixed in 2.5% glutaraldehyde for 2 h at 4 °C, then harvested by centrifugation and washed with 0.1 M PBS. The samples were then postfixed in 1% osmium tetroxide through a series of ethanol dehydration. Finally, the samples were embedded in Durcopan ACM, stained, and imaged by transmission electron microscope (JEM-2000EX; JEOL Co; Japan).

### Immunofluorescence staining for LC3

After being seeded in 24-well plates and treated with different pH (6.4,6.8 and 7.4) for 6 h, cells were rinsed with phosphate-buffered saline (PBS) for three times and fixed with 4% paraformaldehyde for 20 min at room temperature. The samples were then permeabilized with 0.5% TritonX-100 for 20 min and washed three times. Subsequently the samples were incubated with goat serum for 30 min at room temperature. Antibody raised against LC3 was added to every sample and samples were maintained at 4 °C overnight. After being washed with PBST three times, fluorescein-conjugated secondary antibody was added and incubated in dark place for 1 h. DAPI was used to counterstain cell nuclei, and then visualized by microscope.

### Western blot analysis

After OBs were treated with pH 6.4 for 6 h, 12 h and 24 h autophagy marker protein LC3 was detected by Western blot. Cells were washed with cold PBS and lysed with a lysis buffer on the ice. After a series of centrifugations and sample heating, the protein concentrations were measured using the BCA assay. The protein extracts were separated by 15% SDS-polyacrylamide gel and transferred to polyvinylidene difluoride (PVDF) membranes, which had been cut according to the protein band. The membranes were blotted in 5% skim milk for 1 h, then washed and incubated in specific primary antibody LC3 (1:800 dilution) and P62 (1:1000 dilution) overnight at 4 °C. Finally the membrane was put into secondary antibody (1:1000 dilution) for 1 h at room temperature and the protein band was visualized by labwork software.

### Materials

The murine osteoblastic cell line MC3T3-E1 was purchased from Boster (Boster, Wuhan, China). α-minimal essential media (α-MEM) purchased from Hyclone (Logan, Utah, USA). Fetal bovine serum (FBS) was purchased from Gibco (Grand Island, NY, USA). 3-(4,5-Dimethylthiazol-2-yl)-2,5-diphenyltetrazolinum bromide (MTT), 4,6-diamidino-2-phenylindole (DAPI), penicillin, streptomycin, Hepes, chloroquine (CQ), were purchased from Solarbio (Beijing Solarbio Science & Technology, Beijing, China). The primary antibodies against P62, LC3 were purchased from Sigma (St. Louis, MO, USA), β-actin was purchased from ZSGB (ZSGB-BIO, Beijing, China). The Annexin V-FITC apoptosis detection kit was purchased from BD Biosciences (San Diego, CA, USA).

### Statistical analysis

Data are presented as mean ± standard deviation (SD) of three replicates. One-way ANOVA test and t test were employed to compare the difference between experimental groups. SPSS 17.0 software was used to analyse the data. The results were considered significant at P < 0.05.

## Additional Information

**How to cite this article**: Zhang, Z. *et al*. Acidic pH environment induces autophagy in osteoblasts. *Sci. Rep.* **7**, 46161; doi: 10.1038/srep46161 (2017).

**Publisher's note:** Springer Nature remains neutral with regard to jurisdictional claims in published maps and institutional affiliations.
